# miRNome traits analysis on endothelial lineage cells discloses biomarker potential circulating microRNAs which affect progenitor activities

**DOI:** 10.1186/1471-2164-15-802

**Published:** 2014-09-18

**Authors:** Ting-Yu Chang, Tse-Shun Huang, Hsei-Wei Wang, Shing-Jyh Chang, Hung-Hao Lo, Ya-Lin Chiu, Yen-Li Wang, Chung-Der Hsiao, Chin-Han Tsai, Chia-Hao Chan, Ren-In You, Chun-Hsien Wu, Tsung-Neng Tsai, Shu-Meng Cheng, Cheng-Chung Cheng

**Affiliations:** Institute of Microbiology and Immunology, National Yang-Ming University, Taipei, Taiwan; Institute of Engineering in Medicine, UC San Diego, La Jolla, USA; Genome Research Center, National Yang-Ming University, Taipei, Taiwan; Department of Education and Research, Taipei City Hospital, Taipei, Taiwan; Department of Obstetrics and Gynecology, Hsin-Chu Mackay Memorial Hospital, Hsinchu, Taiwan; Department of Bioscience Technology, Chung Yuan Christian University, Chung-Li, Taiwan; Department of Laboratory Medicine and Biotechnology, College of Medicine, Tzu-Chi University, Hualien, Taiwan; Division of Cardiology, Department of Internal Medicine, Tri-Service General Hospital, National Defense Medical Center, Taipei, Taiwan

**Keywords:** Endothelial progenitor cell, smRNA-seq, Circulating microRNA, Coronary artery disease, MicroRNA-221/222

## Abstract

**Background:**

Endothelial progenitor cells (EPCs) play a fundamental role in not only blood vessel development but also post-natal vascular repair. Currently EPCs are defined as early and late EPCs based on their biological properties and their time of appearance during *in vitro* culture. Both EPC types assist angiogenesis and have been linked to ischemia-related disorders, including coronary artery disease (CAD).

**Results:**

We found late EPCs are more mobile than early EPCs and matured endothelial cells (ECs). To pinpoint the mechanism, microRNA profiles of early EPCs late EPCs, and ECs were deciphered by small RNA sequencing. Obtained signatures made up of both novel and known microRNAs, in which anti-angiogenic microRNAs such as miR-221 and miR-222 are more abundant in matured ECs than in late EPCs. Overexpression of miR-221 and miR-222 resulted in the reduction of genes involved in hypoxia response, metabolism, TGF-beta signalling, and cell motion. Not only hamper late EPC activities *in vitro*, both microRNAs (especially miR-222) also hindered *in vivo* vasculogenesis in a zebrafish model. Reporter assays showed that miR-222, but not miR-221, targets the angiogenic factor ETS1. In contrast, PIK3R1 is the target of miR-221, but not miR-222 in late EPCs. Clinically, both miR-221-PIK3R1 and miR-222-ETS1 pairs are deregulated in late EPCs of CAD patients.

**Conclusions:**

Our results illustrate EPCs and ECs exploit unique miRNA modalities to regulate angiogenic features, and explain why late EPC levels and activities are reduced in CAD patients. These data will further help to develop new plasma biomarkers and therapeutic approaches for ischemia-related diseases or tumor angiogenesis.

**Electronic supplementary material:**

The online version of this article (doi:10.1186/1471-2164-15-802) contains supplementary material, which is available to authorized users.

## Background

Defect in angiogenesis or blood vessel repair is the major cause of complications of cardiovascular disorder (CVD) and many ischemia-related diseases, such as diabetes and stroke [1, 2]. Post-natal angiogenesis and vasculogenesis relies, at least partly, on circulating endothelial progenitor cells (EPCs), which may be derived from bone marrow stem cells [3–5]. The regulation of vessel repair depends not only on the number of circulating EPCs but also on the activity of these EPCs [6, 7]. There is a paucity of data examining the EPC status, especially in terms of their functionality, in subjects with metabolic syndrome without diabetes or cardiovascular disease [8]. EPC activities also affect the efficacy of EPC-based transplantation therapy, specifically if only a low number of engraftment migrate to the injured site after infusion, which will limit the clinical applications of EPCs.

Currently EPCs are defined into two distinct populations based on phenotype and biological properties: early EPCs (eEPCs) appear early (<1 week) in culture dishes, whereas late EPCs appear late in culture (2–4 weeks) and have a cobblestone-like morphology [3]. As a result, Yoder’s group denominates late and early EPC as ECFC (colony-forming cell) and CAC (circulating angiogenic cell), respectively [9, 10]. The distinct angiogenic properties of these two EPC subpopulations have been explored using angiogenesis assays: both late EPC and matured endothelial cell (EC), but not early EPC, form capillary microvasculate structures at a similar efficiency [11]. However, early EPC expresses abundant inflammatory cytokines and paracrine angiogenic factors to promote angiogenesis in a paracrine manner [11]. The detailed mRNA expression profiles and functional module analysis for different EPCs have been deciphered [11]. However, detail microRNA (miRNA) expression patterns of early and EPCs are awaited to be explored.

miRNAs are small RNAs of 18–24 nucleotides in length that have emerged as master regulators of angiogenesis [12]. These single-strand small RNAs act by silencing gene expression through imperfect base pairing with cognate transcripts, and one miRNA is able to target multiple different mRNAs since RNA silencing by miRNA does not require perfect sequence complementarity [13]. In the human genome, more than 2500 miRNAs have been discovered (until June 2013, miRBase R20). Tissue miRNAs, as well as circulating miRNAs in body fluids, have recently been considered as a potential approach to identifying new disease biomarkers, and novel drug targets [14–16]. Many pro-angiogenic and anti-angiogenic miRNAs have been pinpointed. Examples like miR-221 and miR-222 are transcribed from the same miRNA cluster and are able to modulate the angiogenic properties of HUVECs by targeting c-Kit and endothelial nitric oxide synthase (eNOS) [17, 18]. Furthermore, levels of EPC miR-221/222 are significantly higher in patients with coronary artery disease (CAD) [19, 20]. Conversely, miR-31-5p is a pro-angiogenic miRNA that induces EPC migration/invasion by targeting FAT4 [21, 22]. miR-10b and miR-196b, which are key regulators of HOX signaling and adult stem cell differentiation, have also been identified as upregulated miRNAs in circulating tumor EPCs and are responsive to vascular endothelial growth factor (VEGF) stimulation [23]. These findings indicate that targeting miRNAs may constitute a novel strategy for manipulating EPC activities for therapeutic purposes.

Up to the present, thousands of miRNAs have been identified in animals and plants, and many more miRNAs are continuously being identified by newly available technologies including small RNA sequencing (smRNA-seq). High-throughput sequencing is not only able to reveal the expression profiles of known miRNAs, but also is able to discover new miRNAs that have not been recorded previously in any databases, in particular the miRBase repository. Small RNA sequencing has been used to carry out research on various types of stem cells, including embryonic stem cells [24–27], hematopoietic stem cells [28], and neural precursor cells [24]. Novel miRNAs have also been identified using smRNA-seq during neural differentiation of embryonic stem cells [27] and during endothelial differentiation [29].

The main goal of this study is to find miRNAs controlling EPC features, hypothesizing that specific miRNAs must be present in cells that have different biological activities. We applied smRNA-seq analyses to endothelial lineage cells for identifying miRNAs that might regulate the activation of the angiogenesis-related phenotypes of EPCs. Novel miRNA-target genes were further identified by tandem array analysis.

## Methods

### Isolation and cultivation of EPCs

Cord blood EPC isolation and characterization were done as described [21, 30]. All patients gave informed consent, and the study was approved by the Mackay Memorial Hospital research ethics committee. The protocols of this study are consistent with the ethical guidelines of the 1975 Helsinki Declaration. In brief, cord blood samples were obtained from healthy donors, and total mononuclear cells (MNCs) were isolated by density gradient centrifugation with Histopaque-1077 (1.077 g/ml, Sigma, MO, USA). MNCs (5 × 10^6^) were plated in 2 ml endothelial growth medium (EGM-2, Lonza, Switzerland) with supplements on fibronectin-coated 6-well plates. After 4 days of culturing, the medium was changed and non-adherent cells were removed, and attached early EPCs appeared to be elongated with spindle shapes. Late EPCs (also known as ECFCs; endothelial colonies forming cells) emerged 2–4 weeks after the start of the MNC culture. For EPC derived from peripheral blood of healthy donors, we collected peripheral blood mononuclear cells (PBMC) from 10 ml whole blood for each individual and followed the same cultivation protocol as cord blood EPC. Late EPCs could be observed 2–4 weeks after the start of PBMC culture and the successful rate of late EPC colony formation was about 50%. The successful rate of late EPC colony formation from peripheral blood of CAD patients was about 10%.

Cloning and transient expression of miR-221/222 expression constructs were described [31]. Cell migration ability was evaluated using Costar Transwell Polycarbonate Permeable Supports (Corning, NY, USA) as previously described [31].

### RNA isolation and quantitative RT-PCR

Total RNA were extracted by the Trizol® solution (Life Technologies, CA, USA). 100 ng to 1 μg of total RNA were subjected into reverse transcription using a First cDNA Synthesis kit (Thermo Fisher Scientific, MA, USA). For quantitative real-time PCR analysis, human pre-messenger RNA sequence was obtained from the NCBI (National Center for Biotechnology Information) AceView program (http://www.ncbi.nlm.nih.gov/IEB/Research/Acembly/). All primers were designed to cross introns using the Primer3 website (http://biotools.umassmed.edu/bioapps/primer3_www.cgi) or Primer Express software (Applied Biosystems, CA, USA). Primers for miRNA qPCR were designed on the basis of stem-loop RT-qPCR for miRNA quantification [32]. Thermodynamics and primer specificity analysis were performed by the Vector NTI suite (Invitrogen, CA, USA) and the NCBI reverse e-PCR program (http://www.ncbi.nlm.nih.gov/sutils/e-pcr/reverse.cgi/). Real-time PCR reactions were performed using Maxima™ SYBR Green qPCR Master Mix (Thermo Fisher Scientific, MA, USA), and the specific products of the PCRs were detected and analyzed using a StepOne™ sequence detector (Applied Biosystems, CA, USA). The expression level of each gene or miRNA was normalized against the expression level of glyceraldehyde 3-phosphate dehydrogenase (GAPDH) or U6 snoRNA. All the primer sequences are listed in Additional file 1: Table S1 online.

### Small RNA sequencing (smRNA-seq) and miRNA data analysis

Small RNA sequencing (smRNA-seq) was done on the Illumina Genome Analyzer IIx platform (GAIIx; Illumina, CA, USA) according to the manufacturer’s instruction. In-house bioinformatics pipelines were constructed for profiling and predicting novel miRNAs from sequencing data [33]. These were firstly the miRNovel pipeline, which was adapted from the miRDeep2 and MIREAP algorithms, and was constructed to predicting and profile novel miRNAs [33]. In this pipeline, Fastq sequences, which were without poly-A tracts, ambiguous nucleotides or a 5′ adapter, but contained the flanking 6–18 nt of the 3′ adapter sequence, had the adapter sequence trimmed and the identical sequences were then collapse to a series of unique sequences. Next, any resulting unique sequences that did not align with the sequences of known mRNAs (UCSC genome browser, http://genome.ucsc.edu/) or known miRNAs (miRBase R20, http://www.mirbase.org/) were aligned to human genome (UCSC genome browser build hg19). Candidate pre-miRNA loci that were predicted by both algorithms were pinpointed and evaluated by manual inspection. We adapted another public available tool HTSeq to quantify the expression values of known miRNAs that were present in the miRBase database (http://www-huber.embl.de/users/anders/HTSeq/doc/overview.html). The expression level of each miRNA was normalized by a RPM (reads per million mapped reads) [34, 35] value calculated as C/MN × 10^9^, where C is “read numbers aligned to a given miRNA chromosomal region”, M is “multiple mapping numbers across all miRNA regions” and N is “total read numbers that map to human genome sequence”.

### Overexpress miR-221 and miR-222 in zebrafish embryos

Sense strand of miR precursors were synthesized *in vitro* by using pCSDEST-derived plasmids as a template. DNA was linearized with *Not*I at 37°C for overnight and cleaned up by DNA Clean/Extraction Kit (GeneMark Inc., Taiwan). Capped mRNA was synthesized by mMESSAGE mMachine SP6 Kit (Life Technologies, CA, USA). Approximately 3 nL *in vitro* synthesized RNA solution was microinjected into the animal pole of one-cell stage embryos of the *Tg(kdrl:EGFP)*^*s843*^ genotype (http://zfin.org/ZDB-GENO-050916-7). The blood vessel phenotype of human miR-injected embryos were scored at 2 days-post fertilization. We quantitated the area of intersegmental blood vessels (ISV) by choosing the same imaging fields of zebra fish embryos and analyzed the positive pixels of ISV by ImageJ software (http://imagej.nih.gov/ij/).

### Statistical analysis

Experiments were repeated for at least 3 times and data are presented as means ± the standard deviation. The Mann–Whitney *U* test was used to compare two groups with small sample size (*p* < 0.05 was considered significant) unless otherwise indicated. Wilcoxon signed-rank test was conducted if the samples are paired.

### Patient information and diagnosis

All patients with coronary artery disease have typical angina and received coronary angiography which showed >50% luminal narrowing in either coronary branches. For the healthy controls, no angina was disclosed and the baseline electrocardiogram did not show myocardial ischemic changes. A summarized table of patient characteristics was listed on Additional file 2: Table S2.

## Results

### Patterns of known miRNAs in different endothelial lineage cells revealed by smRNA-seq

**Figure 1:**
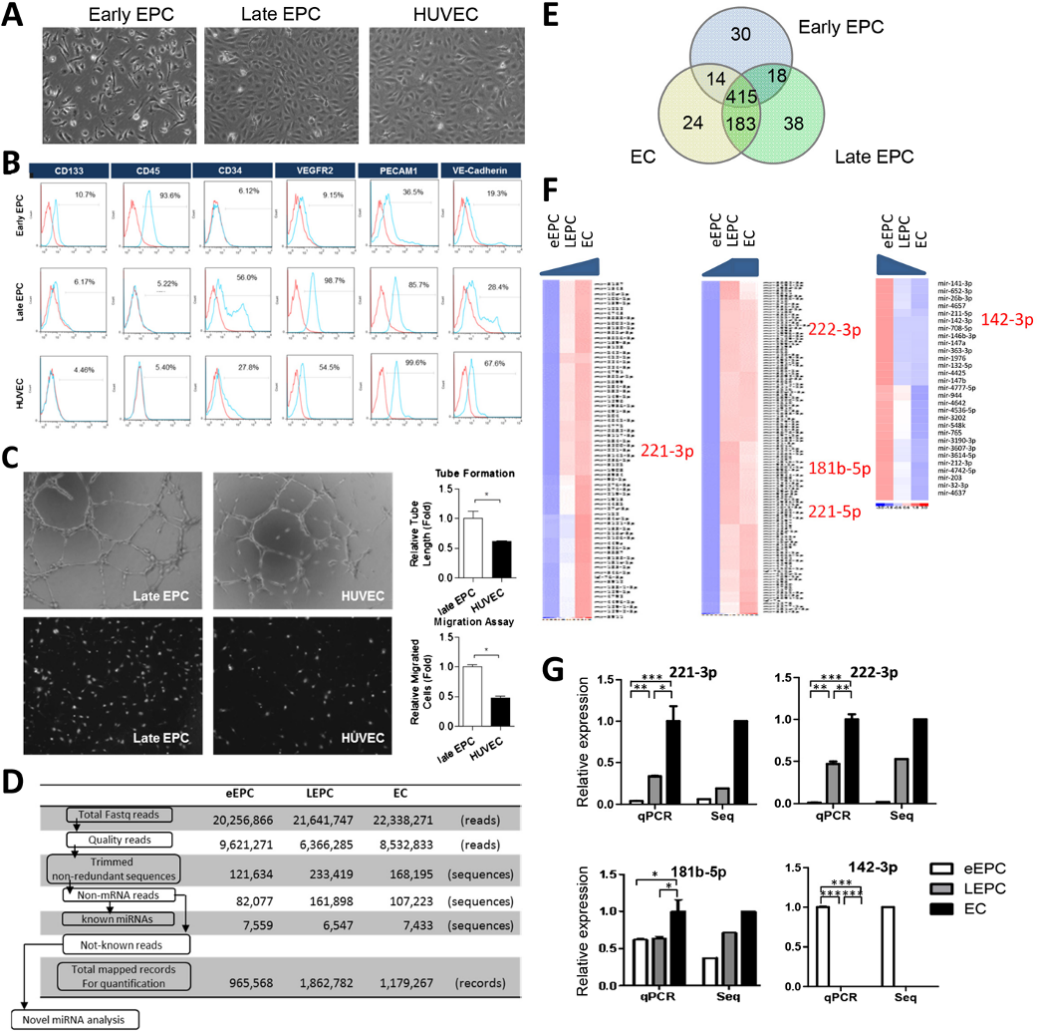
**Interaction network analysis as a framework for the interpretation of EPC biology. (A)** Cord blood mononuclear cells were isolated and plated on fibronectin-coated culture dishes for four days. Adherent early EPCs are shown. Twenty-one days after plating, late EPCs with a cobblestone-like morphology were selected, reseeded, and grown to confluence. **(B)** Expression of progenitor, endothelial and hematopoietic markers on EPCs and HUVEC by flow cytometry analysis. Cyan: fluorescent signal using indicated antibodies; Red: isotype control. **(C)** Tube formation (*upper*) and Transwell (*lower*) assays of late EPC and HUVEC. Right panels: quantitative results of the two assays (n = 3) *: *p* < 0.05. **(D)** The analysis pipeline for identification of known miRNAs from smRNA-seq data. Reads or sequences pass each filtration process were indicated. **(E)** Venn diagram of expressed known miRNAs (RPM > 100). **(F)** Heat maps of known miRNA expression profiles. Left: EC > late EPC > early EPC at a 1.5 fold change (FC); Middle: FC between late EPC and EC < 1.5, which between late EPC and early EPC > 1.5; Right: early EPC > late EPC > HUVEC at a 1.5 fold change. Blue, downregulated; Red, upregulated. **(G)** RT-qPCR confirmation of selected miRNAs. White bars: early EPC; grey: late EPC; black: mature EC. Histograms of qPCR results were graphed as mean ± standard deviation (n = 3) *: *p* < 0.05, **: *p* < 0.01, ***: *p* < 0.001.

Early and late EPCs were obtained from the cord blood of healthy subjects as described [30], and matured ECs were isolated from the umbilical cord vein of the same donors. Blood mononuclear cells were round when initially seeded on the fibronectin-coated wells. After changing the medium on day 4, attached eEPCs with an elongated morphology could be observed (Figure 1A). Late EPCs with a cobblestone-like morphology, which is similar to mature ECs, had grown to confluence by day 14 to day 21 (Figure 1A, the “Late EPC” and “HUVEC” panels).

Pilot EPC and EC characterization was performed by flow cytometry: the stem cell marker CD133 is expressed in ~10% of early EPCs [36], and precursor marker CD34 is more abundant in late EPCs (Figure 1B). The hematological cell marker CD45 was present in early EPCs but not late EPCs or HUVECs (Figure 1B). The endothelial marker CD31 (PECAM-1, platelet/endothelial cell adhesion molecule-1) is expressed well in early EPCs, late EPCs, and HUVECs (Figure 1B). Other endothelial markers, including VE-Cadherin and KDR/VEGFR2 could be identified in late EPCs and HUVECs (Figure 1B). Of note, VEGFR2 is more abundant in late EPCs (Figure 1B).The abundant expression of the precursor marker CD34 and the angiogenic receptor VEGFR2 raised one possibility that late EPCs, due to their relatively undifferentiated status, may be and more active than matured ECs. This possibility was examined by comparing the cell migration and microtubule formation abilities between late EPCs and HUVEC. Transwell and MatriGel tube formation assays showed that these angiogenesis-related activities are livelier in late EPCs (Figure 1C).

To explore the underlying mechanisms, miRNA expression patterns in different EPCs and matured HUVEC were deciphered by smRNA-seq. The analysis pipeline used to identify known miRNAs, namely those that have been deposited in the miRBase database R20, is shown in Figure 1D, and a total of 722 miRBase miRNAs were expressed in these three endothelial lineage cells (Figure 1E). Among them, 69 and 29 miRNAs were enriched or repressed, respectively, in HUVEC in a monotonic pattern (≥1.5 fold change; left and right panels in Figure 1F, details in Additional file 3: Table S3 online), and 124 microRNAs were more abundant in late EPCs/HUVEC than in early EPCs (middle panel of Figure 1F & Additional file 3: Table S3). RT-qPCR using independent batches of cord blood EPCs verified that anti-angiogenic miRNAs such as miR-221-3p and miR-222-3p (was known as miR-221 and miR-222) [17, 18] were enriched in matured ECs (Figure 1G). miR-181b-5p, which inhibits arsenic-induced endothelial cell migration/tube formation and NF-κB-mediated EC activation and inflammation [37, 38], is also more abundant in HUVEC (Figure 1G).

**Figure 2:**
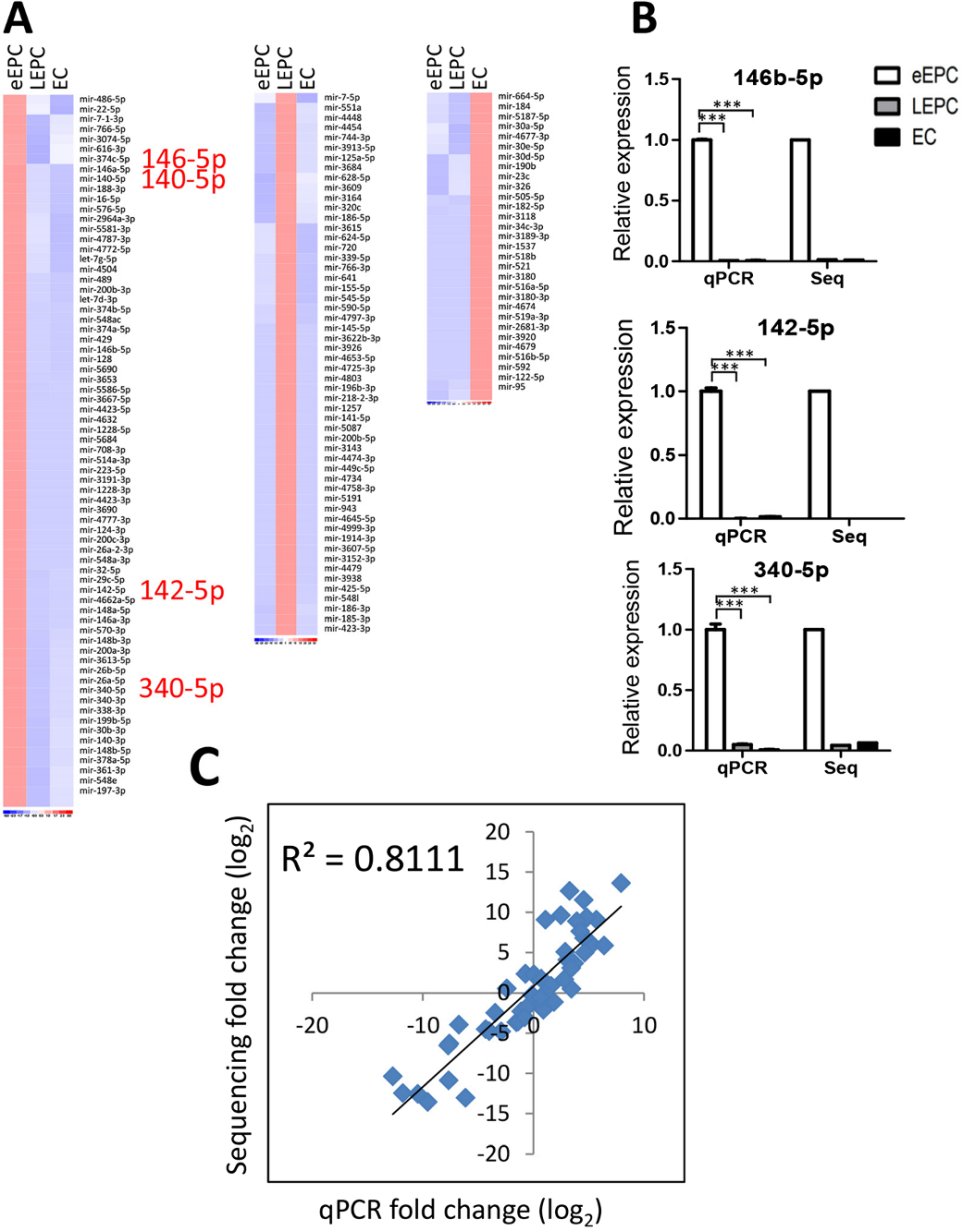
**Validation of smRNA-seq data. (A)** Heat maps of miRNAs dominantly expression in early EPC (left), late EPC (middle), and mature EC (right) with at least a 1.5 fold change. **(B)** RT-qPCR (qPCR) validation of smRNA-seq (Seq) results. Histograms of qPCR results were graphed as mean ± standard deviation (n = 3) ***: *p* < 0.001. **(C)** Pearson’s correlation result between smRNA-seq and qPCR data.

Twenty-four miRNAs were specific expressed in HUVEC, 38 in late EPCs, and another 30 in early EPCs (Figure 1E and Additional file 4: Table S4). There expression profiles were summarized by heatmaps in Figure 2A. RT-qPCR assays again validated the unique expression of miR-146-5p, miR-143-5p and miR-340-5p in early EPCs (Figure 2B). Our sequencing results are reliable since they correlated well with RT-qPCR validation data (R^2^ = 0.811; Figure 2C).

### Novel EPC and/or EC miRNAs identified by smRNA-seq

**Figure 3:**
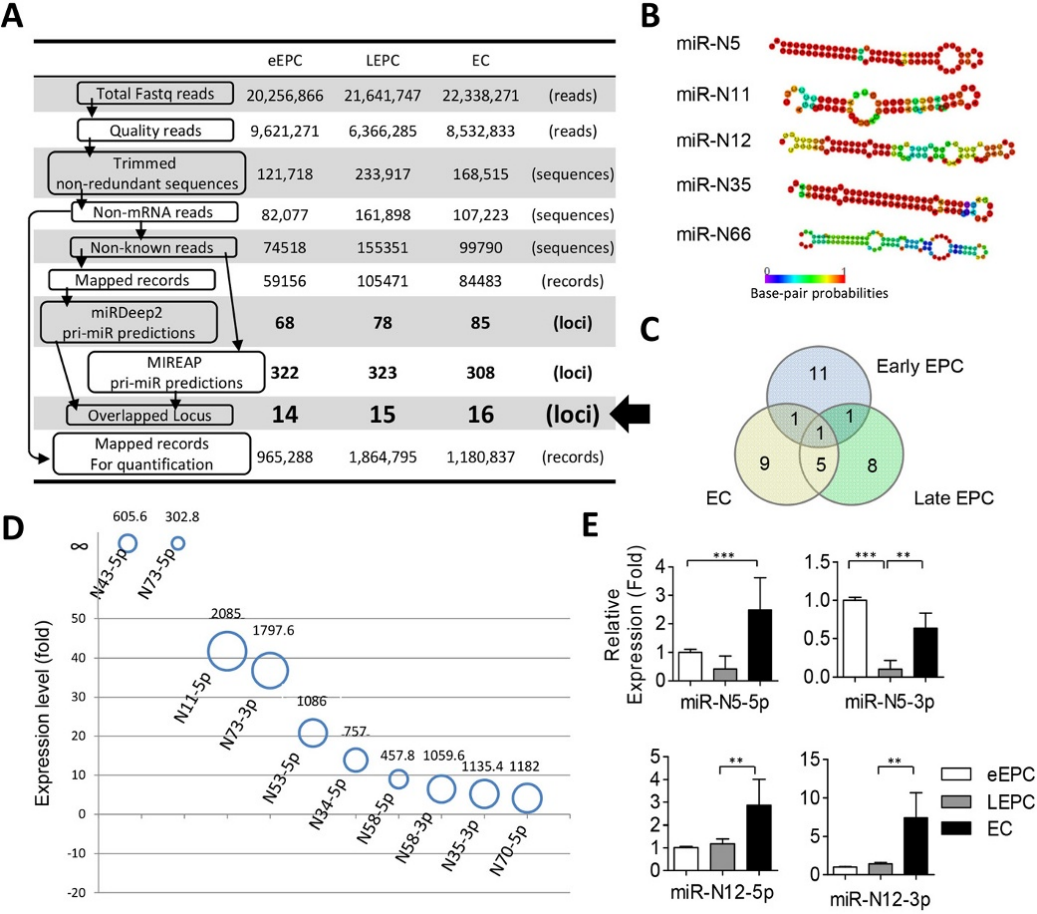
**smRNA-seq reveals novel miRNAs in endothelial lineage cells. (A)** A flowchart describes the data analysis pipeline. The numbers of sequences and reads remaining at each step of the data analysis are indicated. Reads numbers include sequences map to more than one locus in the genome. **(B)** Deduced RNA secondary structures of a set of newly discovered miRNAs. **(C)** A Venn diagram showing unique and common novel miRNAs in each cell type. **(D)** The top ten differentially expressed novel miRNAs between late EPC and other cell types. Areas and numbers above the circles indicate miRNA RPM values in late EPCs. **(E)** Validation of smRNA-seq data by RT-qPCR. All histograms were graphed as mean ± standard deviation (n = 3) **: *p* < 0.01, ***: *p* < 0.001.

smRNA-seq rather than microarrays for miRNome research is that sequencing technology can not only profile known miRNA profiles but also help to pinpoint novel and previous unidentified miRNAs. Sequencing results generated 6.4 to 9.6 million high-quality reads that corresponded to 120,000 to 234,000 non-redundant small RNA sequences (Figure 3A). A data analysis pipeline [33] was applied to remove mRNA contamination and collect the reads not mapped to any known miRNA loci. Novel miRNAs predicted by both of the two independent bioinformatics algorithms (miRDeep2 and MIREAP, Figure 3A) were recognized as candidate novel miRNAs. These data processing steps yielded 14 ~ 16 unique genomic loci that potentially encoded novel miRNAs in either cell type (Figure 3A, indicated by an arrow).

All these putative novel miRNAs had the potential to fold into a hairpin secondary structure, which is one of the criteria necessary for miRNAs. Figure 3B illustrates some examples. Among these candidates, eleven novel miRNAs were uniquely expressed in early EPCs, eight in late EPCs, while another nine in matured ECs (Figure 3C). Additional file 5: Table S5 and Figure 3D illustrates the abundance, sequences and chromosomal coordinates of these novel miRNAs (temporally designated as miR-N), as well as cell type distributions. The existence of four novel miRNAs (N5-3p/-5p and N12-3p/-5p) in EPCs and ECs was verified by RT-qPCR (Figure 3E).

### *miR-221/222*cluster miRNAs contribute to EPC motility and blood vessel formation

**Figure 4:**
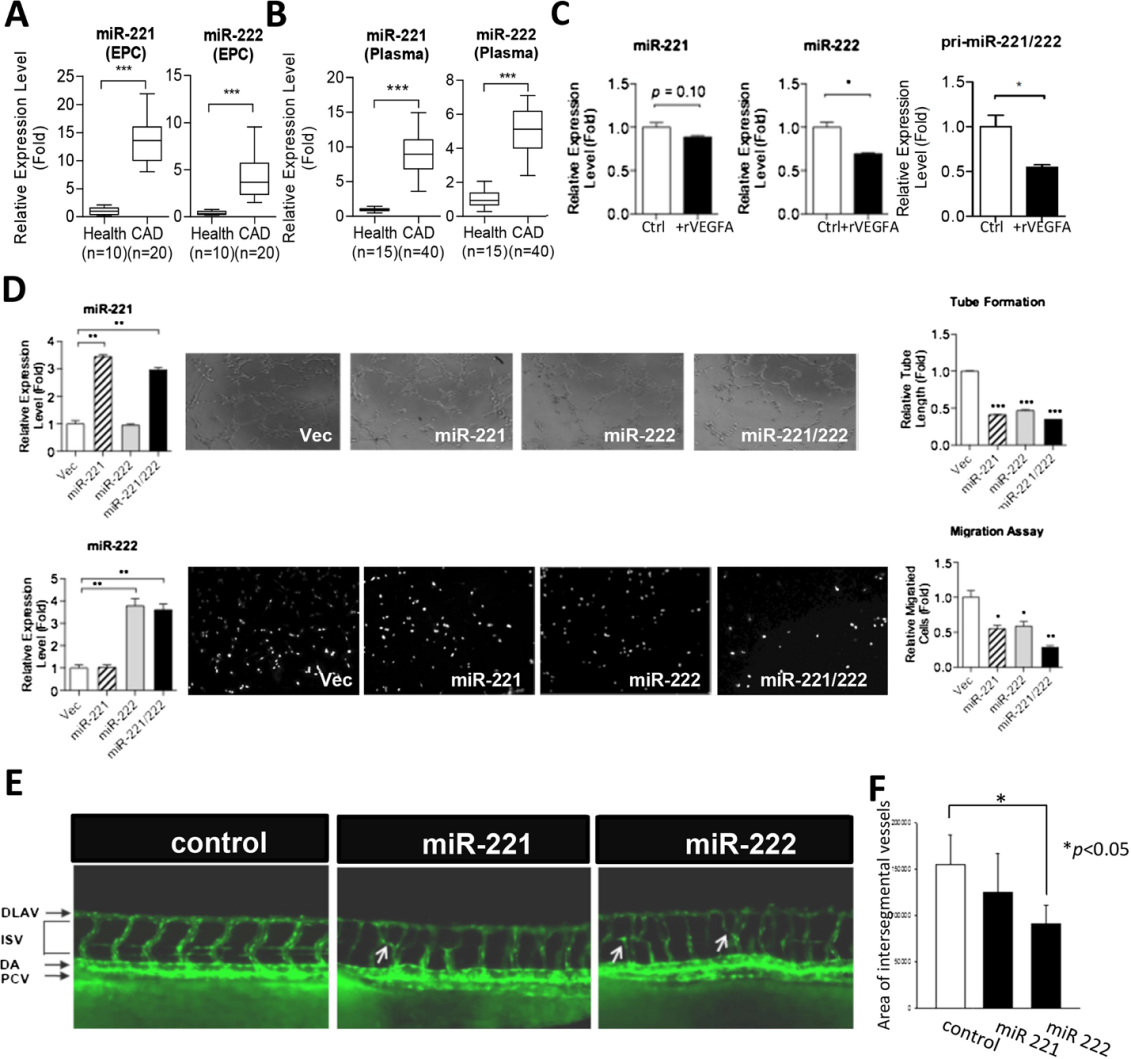
**Decreased miR-221/222 levels in CAD patients and the contribution of miR-221/222 in late EPC functions. (A-B)** RT-qPCR results show miR-221/222 expression levels in late EPCs (*A*) and plasma (*B*) from health controls and CAD patients. ***: *p* < 0.0001 by Mann–Whitney test. **(C)** RT-qPCR results of miR-221 (left), miR-222 (middle) and pri-miR-221/222 (right) levels in late EPCs treated with recombinant VEGFA (n = 3). *: *p* < 0.05. **(D)** Tube formation (upper) and Transwell migration (lower) assays were performed in late EPCs transfected with indicated miRNAs for evaluation of endothelial functions. Left histograms: RT-qPCR of miRNA expression levels; Right histograms: quantitative results of tube formation and migration assays (n = 3). **: *p* < 0.01, ***: *p* < 0.001. **(E-F)** Overexpression of miR-221 or miR-222 in zebrafish embryos causes abnormal blood vascular development. Embryos in the Tg(kdrl:EGFP)^s843^ background were injected with 460 pg of negative control RNA (n = 100), 560 pg RNA of miR-221 (n = 106), or 560 pg RNA of miR-222 (n = 62). Blood vessel patterns were observed (*E*) and quantified (*F*) 48 hours postfertilization (hpf). DLAV: dorsal longitudinal anastomotic vessel; ISV: intersegmental vessels; DA: dorsal aorta; PCV: posterior cardinal vein.

Consistent with the less active nature of HUVEC (Figure 1C), two anti-angiogenic miRNAs, miR-221 and miR-222, were more abundant in matured ECs (Figure 1G). It has been reported that levels of miR-221 and miR-222 are higher in EPCs from patients with coronary artery disease (CAD) [19, 20], but the anti-angiogenic role of miR-221/222 and the downstream mechanism in late EPCs is unclear. We verified that late EPCs isolated from the peripheral blood of CAD patients expressed more miR-221/222, especially miR-221, than those from healthy controls (Figure 4A). More significantly, levels of circulating miR-221/222 in the plasma of CAD patients were also higher than those in healthy controls (Figure 4B, *p* < 0.0001). We also tested the effect of VEGF on the expression of miR-221/222 to examine whether they are downregulated in an angiogenesis-promoting environment. VEGF repressed the levels of matured miR-222 and pri-miR-221/222 in diseased late EPCs (Figure 4C). However, miR-221 levels were not significantly affected by VEGF (Figure 4C), indicating a post-transcriptional event may also be involved in the regulation of miR-221 levels in CAD late EPCs.

Overexpressing miR-221/222 reduced the *in vitro* cellular motility and microvasculature formation ability of late EPCs (Figure 4D), which partly explained why these 2 miRs were more abundant in disease EPCs and in mature ECs. Since EPCs are capable of forming new blood vessels even in the absence of a pre-existing vessel network [6] and knockdown experiments showed that miR-221 is required for endothelial tip cell behaviors during vascular development [39], we examined the role of miR-221/222 in the formation of the blood vessels *in vivo*. Over-expression of either miR-221 or miR-222 in zebrafish embryos resulted in the deregulation of blood vessel pattern during development (Figure 4E, abnormal blood vessels indicated by arrows). miR-222, but not miR-221, led to a significant reduction in blood vessel density *in vivo* (Figure 4F).

### miR-221/222 affect genes involved in hypoxia response, cell migration, energy supply and so on

**Figure 5:**
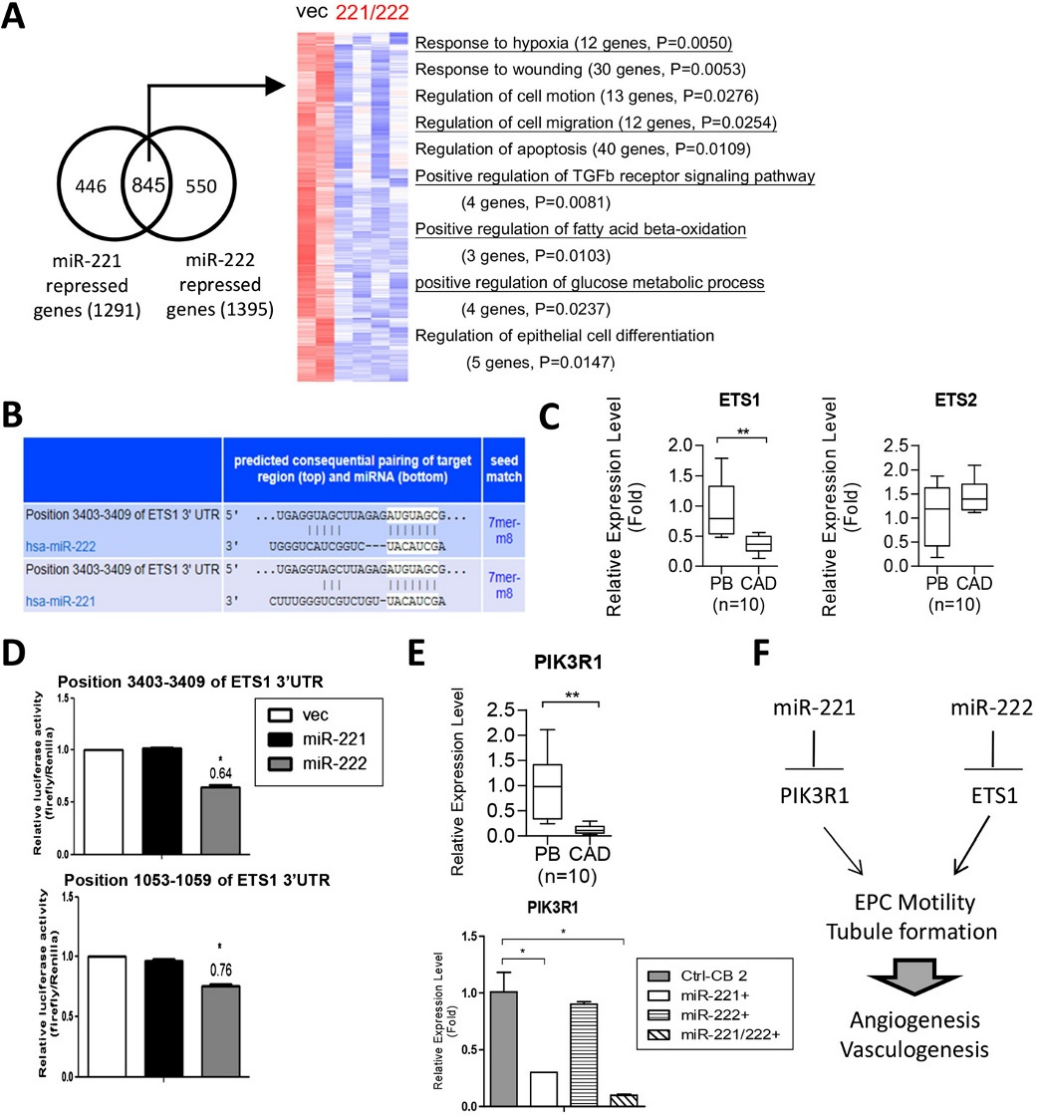
**miR-221 and miR-222 regulate PIK3R1 and ETS1, respectively, in late EPCs. (A)** A Venn diagram illustrates there were 845 probe sets commonly repressed by miR-221 and miR-222 (*left*). (*right*) A heat map showing the relative expression levels of these genes. Significant enriched biological processes among these genes according to the Gene Ontology database were also indicated. **(B)** Predicted duplex formed between ETS1 and miR-222/miR-221. **(C)** RT-qPCR results show ETS1 (*left*) and ETS2 (*right*) expression levels in late EPCs from health controls (PB) and CAD patients. **: *p* < 0.01 by Mann–Whitney test. **(D)** Reporter activity of the ETS1 on two 3′UTR reporter constructs containing the conserved binding site (*upper*) and poorly conserved binding site (*lower*) after cotransfection with the miR-221, miR-222 or empty vector into 293 T cells (n = 3). **(E)** miR-221 targets PIK3R1 in late EPCs. RT-qPCR show the levels of PIK3R1 in health and CAD late EPCs (*upper*), or in late EPCs overexpressed with miR-221. miR-222, or both (*lower*) (n = 3). **(F)** A model of miR-221 and miR-222 regulate EPC motility and tube formation functions through PIK3R1 and ETS1, respectively.

To explore the underlying mechanisms, we aligned genes down-regulated by miR-221 and miR-222. A total of 845 genes were commonly repressed by both miRNAs (Figure 5A). Gene ontology analysis showed that genes involved in hypoxia response, cell migration, energy supply from glucose or fatty acids metabolism, and TGF-beta receptor signaling pathway were selectively repressed by both miRs (Figure 5A, underlined), consistent with the anti-angiogenic role of these two functional-related miRNAs. Twelve cell migration genes (AGT, CITED2, CXCR4, F2RL1, F3, HDAC6, IRS2, IGFBP3, PTEN, PIK3R1, PTPRM, & TGFBR3) were reduced in the presence of miR-221/222, consistent with the reduced cell motility phenotype in Figure 4D. Repression of CXCR4 levels by both miR-221 and miR-222 was confirmed by RT-qPCR (Additional file 6: Figure S1).

Nevertheless, no miR-221- or miR-222-binding site could be identified on CXCR4 mRNA (not shown), we searched for direct targets for either miRNAs. We found ETS1 is among miR-222-suppressed genes in late EPCs and possesses miR-221/-222 binding sites on its 3′UTR according to the TargetScan bioinformatics prediction (Figure 5B). Since miR-221 and miR-222 are known to target ETS2 and ETS1, respectively, in matured blood vessel endothelial cells [31], we tested whether ETS1 and ETS2 levels in late EPCs from CAD patients. Only ETS1, but not ETS2, were differentially expressed between diseased and healthy late EPCs (Figure 5C). The direct repression of ETS1 translation by miR-221 and miR-222 was examined by 3′UTR reporter assays. There are two miR-221/222 binding sites on 3′UTR of ETS1 (NM_005238) predicted by TargetScanHuman (http://www.targetscan.org/vert_61/), one is conserved (3403–3409 of ETS1 3′UTR); another is poorly conserved (1053–1059 of ETS1 3′UTR.) Only miR-222, but not miR-221, inhibited ETS1 translation through both putative binding sites (Figure 5D). Reporter assays on mutated ETS1 3′-UTR to show that the miR-222 binding site is functional have been done [31].

We also searched target(s) for miR-221 in CAD late EPCs. It is known that miR-221 promotes tip cell behavior during vascular development through the repression of phosphoinositide-3-kinase regulatory subunit 1 (PIK3R1) [39]. PIK3R1 levels were reduced in diseased late EPCs (Figure 5E, upper panel). When miR-221 and miR-222 were overexpressed in late EPCs, only miR-221, but not miR-222, repressed PIK3R1 (Figure 5E, lower panel).

## Discussion

Circulating miRNAs have attracted major interest as biomarkers for cardiovascular diseases [40]. Besides their cell intrinsic function, recent studies reported that miRNAs are released by cells and can be detected in the blood [41]. Here we identified the miRNome profiles of early EPC, late EPC and HUVEC of the same donor. Late EPC was found to possess the best angiogenic activities and less miR-221/222 expression. Furthermore, circulating miR-221 and miR-222 levels in the plasma of CAD patients were found to be higher. Accordingly, other EPC and EC miRNAs revealed in this study may also hold the potential of being new biomarkers in plasma for monitoring CAD or CAD-like cases among high risk population (such as the metabolic syndrome population) (Additional file 3: Table S3).

Human EPCs research these years is quite active, partly due to the clinical application potentials of these cells. The angiogenic abilities as well as homing of transplanted EPCs to injured sites are critical parameters for therapy to be successful. The number of circulating EPCs in a patient’s peripheral blood correlates with the prognosis of many diseases, such as cancer, diabetes, stroke, and CVD [42, 43]. These cells are also potential sources for cell therapy that aims to enhance the neovascularization of tissue engineered constructs or ischemic tissues [44]. Many studies evaluated the possibility of transplantation of late EPC for improving disease recovery, for example, the long-term stroke outcome in a mouse model of transient middle cerebral artery occlusion (MCAO) [44, 45]. Angiogenic activities as well as homing of transplanted EPCs to injured sites are therefore critical parameters for therapy to be successful. Genomic tools, including transcriptomic and proteomic ones, have been applied to decipher the gene expression profiles of early and late EPCs for understanding and manipulating genes in EPCs for therapeutic purposes [45, 46]. We recently pinpointed novel surface biomarkers and stemness genes in different EPCs, as well as between EPCs and matured ECs [11]. With this information, it is possible to sort EPCs from circulation as well as to identify molecular targets crucial for EPC functioning during wound healing and tumor angiogenesis. Here we further weighted in this field by disclosing miRNA profiles in different EPCs and mature ECs. More in-depth wetlab work is still necessary to explore genes and miRNA responsible for EPC or EC activities.

RNA-seq, an application of the emerging ultrahigh throughput next generation sequencing (NGS) technologies, has become an attractive alternative to microarrays. Not only does it allow researchers to identify differentially expressed genes, RNA-seq can further identify novel transcripts that have not yet been recorded in any literatures or databases [47]. The novelty of this study is also that we pinpointed a significant number of novel miRNAs that vary across EPCs and ECs (Figure 3 and Additional file 5: Table S5). These findings show that the full repertoire of miRNAs in endothelial lineage cells is larger and more complicated than previously appreciated. Unmasking the various roles of newly identified miRNAs in EPC/EC activity and endothelial differentiation will provide new insights for regenerative medicine. We envision our report will serve as a resource and road map for future studies that are aimed at improving our understanding of the various regulatory pathways that ultimately modulate EPC/EC activities and angiogenesis.

Clinical trials using bone marrow-derived CD34+ or CD133+ stem cells have reported only marginal benefits for treating CAD patients [39, 48, 49]. Goretti *et al.* demonstrated that miR-16 family (miR-15a/-15b/-16/-103/-107) which overexpressed in early EPCs may restrict the vessel repair function of EPCs through inhibition of cell cycle progression associated gene CCND1, CCNE1 and CDK6 [36]. Compared with their results, which were performed by microarray screening, we also identified similar miRNAs up- or down-regulated in early EPC (up: let-7 g-5p, miR-16-5p, miR-26b-5p, miR-30b-3p, miR-140-5p, miR-146a-5p, miR-146a-3p and miR-338-3p) or in late EPC (miR-27a-3p, miR-27b-5p, miR-27b-3p, miR-151a-5p and miR-193a-5p). Nevertheless, Goretti *et al.* did not include mature ECs in their comparison. Here we used 3 endothelial lineage cell types so provide another level of information. Cell type-specific miRs will be changed accordingly. Furthermore, owing to the advanced smRNA-seq technique, we identified more miRNAs than the traditional microarray strategy did.

miR-221 and miR-222 are two highly homologous miRNAs encoded in tandem from human chromosome Xp11.3 and are highly conserved in vertebrates. We observed defective vascular growth when miR-221 or miR-222, especially miR-222, was overexpressed in zebrafish. Overexpressing miR-221 in zebrafish caused deregulated blood vessel pattern but not the reduction of vessel density, which may partly due to the fact that miR-221 is required for vascular development by promoting tip cell migration and proliferation in zebrafish during vascular development [39]. miR-221 and miR-222 also inhibit erythropoiesis and erythroleukemic cell growth *via* down-modulating cKit [50]. miR-221/222 inhibit EC [31] and EPC motility and microtubule formation. Consistent with these observations, in late EPCs from CAD patients the expression levels of miR-221 and miR-222 are increased (Figure 4A) [20]. Reduced levels of circulating EPCs independently predict atherosclerotic disease progression and development of cardiovascular events [7]. Clinical studies demonstrated that levels of circulating EPCs are associated with vascular endothelial function and cardiovascular risk factors, and help to identify patients at increased cardiovascular risk [8]. Circulating EPCs in peripheral blood are mobilized from bone marrow by chemokine stimulation such as chemokine (C-X-C motif) ligand 12 (CXCL12; also known as stromal derived factor-1, SDF-1) [51]. We found both miR-221 and miR-222 repressed in late EPCs the levels of chemokine (C-X-C motif) receptor 4 (CXCR4), the receptor for CXCL12 (Figure 4), partly explains why miR-221/222 inhibit late EPC motility (Figure 4C), and EPC levels were reduced in the peripheral blood of CAD patients. Since neither miR-221 nor miR-222 targets CXCR4 directly according to bioinformatics prediction, how CXCR4 levels are regulated is still an open question. We demonstrated another candidate miR-221 target gene – PIK3R1, which is also important in regulating endothelial tip cell migration and proliferation in EPC [39]. PIK3R1 is the p85α regulatory subunit of phosphoinositide-3-kinase (PI3K). Previous study indicated miR-221 may regulate the delicate stoichiometry of PIK3R1 subunit to maintain the correct subcellular localization and regulate downstream *flt4* expression [39]. ETS1 is a known target of miR-222 in mature endothelial cells [31] and contributes to embryonic angiogenesis regulation and may play a role in the modulation and maturation of EPC. miR-221 and miR-222 each has unique targets and different mechanisms to repress the activities of diseased late EPCs (Figure 5F). Other biomarker candidates among our mined miRNAs with supporting references are indicated in Additional file 3: Tables S3.

## Conclusions

In summary, our results demonstrate miR-221 and miR-222 repress the levels of PIK3R1 and ETS1, respectively to regulate angiogenic features in EPCs and ECs (Figure 5F) and suggest mechanisms of why late EPC levels and activities are reduced in CAD patients. Overall, our data provide evidences that miRNA research will not only help us to understand more about EPC biology, but will also lead to the development of novel biomarkers and therapeutic modalities for the prevention and treatment of CAD and other ischemia-related diseases such as stroke and diabetes.

## Electronic supplementary material

Additional file 1: Table S1: Oligo sequences. (XLS 30 KB)

Additional file 2: Table S2: Baseline characteristic of 55 studied subjects in healthy and CAD patients. (XLS 32 KB)

Additional file 3: Table S3: miRNA expression profile in Figures 1F and 2A. (XLS 95 KB)

Additional file 4: Table S4: Stage specific miRNA expression profile. (XLS 120 KB)

Additional file 5: Table S5: Predicted novel miRNAs. (PPTX 114 KB)

Additional file 6: Figure S1: RT-qPCR validation showing the repression of CXCR4 levels in late EPCs overexpressing with miR-221 or miR-222 (n=3). (PDF 145 KB)
