# Supplementary material for: miRNome traits analysis on endothelial lineage cells discloses biomarker potential circulating microRNAs which affect progenitor activities
Source: BMC Genomics. 2014 Sep 18;15(1):802. doi: 10.1186/1471-2164-15-802 (PMC4176563; doi:10.1186/1471-2164-15-802)
Supplement: Supplementary file 5 — Additional file 5: Table S5: Predicted novel miRNAs. (PPTX 114 KB) [file 12864_2014_6478_MOESM5_ESM.pptx]

## Slide 1
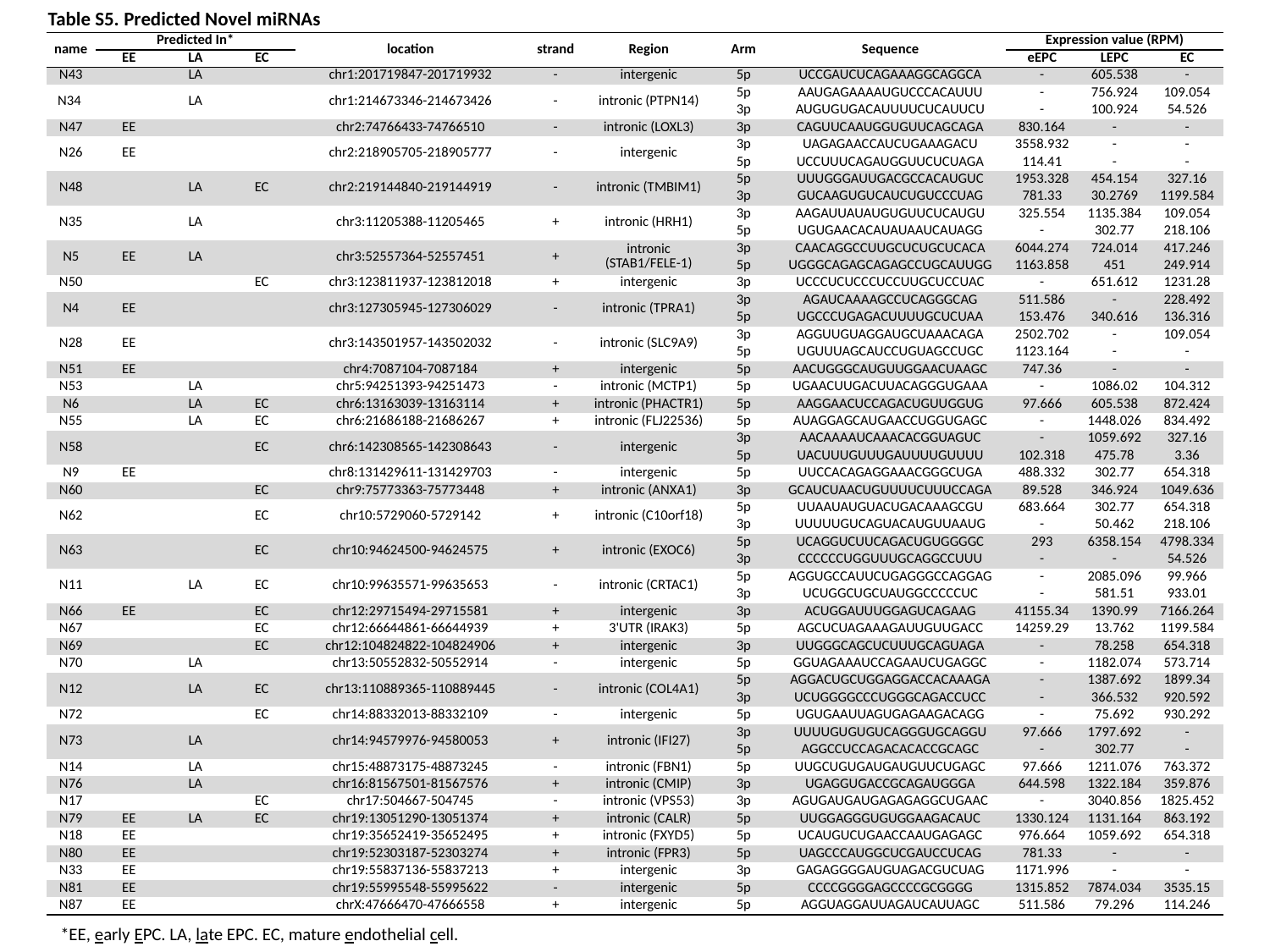

Table S5. Predicted Novel miRNAs
| name | Predicted In\* | | | location | strand | Region | Arm | Sequence | Expression value (RPM) | | |
| --- | --- | --- | --- | --- | --- | --- | --- | --- | --- | --- | --- |
| | EE | LA | EC | | | | | | eEPC | LEPC | EC |
| N43 | | LA | | chr1:201719847-201719932 | - | intergenic | 5p | UCCGAUCUCAGAAAGGCAGGCA | - | 605.538 | - |
| N34 | | LA | | chr1:214673346-214673426 | - | intronic (PTPN14) | 5p | AAUGAGAAAAUGUCCCACAUUU | - | 756.924 | 109.054 |
| | | | | | | | 3p | AUGUGUGACAUUUUCUCAUUCU | - | 100.924 | 54.526 |
| N47 | EE | | | chr2:74766433-74766510 | - | intronic (LOXL3) | 3p | CAGUUCAAUGGUGUUCAGCAGA | 830.164 | - | - |
| N26 | EE | | | chr2:218905705-218905777 | - | intergenic | 3p | UAGAGAACCAUCUGAAAGACU | 3558.932 | - | - |
| | | | | | | | 5p | UCCUUUCAGAUGGUUCUCUAGA | 114.41 | - | - |
| N48 | | LA | EC | chr2:219144840-219144919 | - | intronic (TMBIM1) | 5p | UUUGGGAUUGACGCCACAUGUC | 1953.328 | 454.154 | 327.16 |
| | | | | | | | 3p | GUCAAGUGUCAUCUGUCCCUAG | 781.33 | 30.2769 | 1199.584 |
| N35 | | LA | | chr3:11205388-11205465 | + | intronic (HRH1) | 3p | AAGAUUAUAUGUGUUCUCAUGU | 325.554 | 1135.384 | 109.054 |
| | | | | | | | 5p | UGUGAACACAUAUAAUCAUAGG | - | 302.77 | 218.106 |
| N5 | EE | LA | | chr3:52557364-52557451 | + | intronic (STAB1/FELE-1) | 3p | CAACAGGCCUUGCUCUGCUCACA | 6044.274 | 724.014 | 417.246 |
| | | | | | | | 5p | UGGGCAGAGCAGAGCCUGCAUUGG | 1163.858 | 451 | 249.914 |
| N50 | | | EC | chr3:123811937-123812018 | + | intergenic | 3p | UCCCUCUCCCUCCUUGCUCCUAC | - | 651.612 | 1231.28 |
| N4 | EE | | | chr3:127305945-127306029 | - | intronic (TPRA1) | 3p | AGAUCAAAAGCCUCAGGGCAG | 511.586 | - | 228.492 |
| | | | | | | | 5p | UGCCCUGAGACUUUUGCUCUAA | 153.476 | 340.616 | 136.316 |
| N28 | EE | | | chr3:143501957-143502032 | - | intronic (SLC9A9) | 3p | AGGUUGUAGGAUGCUAAACAGA | 2502.702 | - | 109.054 |
| | | | | | | | 5p | UGUUUAGCAUCCUGUAGCCUGC | 1123.164 | - | - |
| N51 | EE | | | chr4:7087104-7087184 | + | intergenic | 5p | AACUGGGCAUGUUGGAACUAAGC | 747.36 | - | - |
| N53 | | LA | | chr5:94251393-94251473 | - | intronic (MCTP1) | 5p | UGAACUUGACUUACAGGGUGAAA | - | 1086.02 | 104.312 |
| N6 | | LA | EC | chr6:13163039-13163114 | + | intronic (PHACTR1) | 5p | AAGGAACUCCAGACUGUUGGUG | 97.666 | 605.538 | 872.424 |
| N55 | | LA | EC | chr6:21686188-21686267 | + | intronic (FLJ22536) | 5p | AUAGGAGCAUGAACCUGGUGAGC | - | 1448.026 | 834.492 |
| N58 | | | EC | chr6:142308565-142308643 | - | intergenic | 3p | AACAAAAUCAAACACGGUAGUC | - | 1059.692 | 327.16 |
| | | | | | | | 5p | UACUUUGUUUGAUUUUGUUUU | 102.318 | 475.78 | 3.36 |
| N9 | EE | | | chr8:131429611-131429703 | - | intergenic | 5p | UUCCACAGAGGAAACGGGCUGA | 488.332 | 302.77 | 654.318 |
| N60 | | | EC | chr9:75773363-75773448 | + | intronic (ANXA1) | 3p | GCAUCUAACUGUUUUCUUUCCAGA | 89.528 | 346.924 | 1049.636 |
| N62 | | | EC | chr10:5729060-5729142 | + | intronic (C10orf18) | 5p | UUAAUAUGUACUGACAAAGCGU | 683.664 | 302.77 | 654.318 |
| | | | | | | | 3p | UUUUUGUCAGUACAUGUUAAUG | - | 50.462 | 218.106 |
| N63 | | | EC | chr10:94624500-94624575 | + | intronic (EXOC6) | 5p | UCAGGUCUUCAGACUGUGGGGC | 293 | 6358.154 | 4798.334 |
| | | | | | | | 3p | CCCCCCUGGUUUGCAGGCCUUU | - | - | 54.526 |
| N11 | | LA | EC | chr10:99635571-99635653 | - | intronic (CRTAC1) | 5p | AGGUGCCAUUCUGAGGGCCAGGAG | - | 2085.096 | 99.966 |
| | | | | | | | 3p | UCUGGCUGCUAUGGCCCCCUC | - | 581.51 | 933.01 |
| N66 | EE | | EC | chr12:29715494-29715581 | + | intergenic | 3p | ACUGGAUUUGGAGUCAGAAG | 41155.34 | 1390.99 | 7166.264 |
| N67 | | | EC | chr12:66644861-66644939 | + | 3'UTR (IRAK3) | 5p | AGCUCUAGAAAGAUUGUUGACC | 14259.29 | 13.762 | 1199.584 |
| N69 | | | EC | chr12:104824822-104824906 | + | intergenic | 3p | UUGGGCAGCUCUUUGCAGUAGA | - | 78.258 | 654.318 |
| N70 | | LA | | chr13:50552832-50552914 | - | intergenic | 5p | GGUAGAAAUCCAGAAUCUGAGGC | - | 1182.074 | 573.714 |
| N12 | | LA | EC | chr13:110889365-110889445 | - | intronic (COL4A1) | 5p | AGGACUGCUGGAGGACCACAAAGA | - | 1387.692 | 1899.34 |
| | | | | | | | 3p | UCUGGGGCCCUGGGCAGACCUCC | - | 366.532 | 920.592 |
| N72 | | | EC | chr14:88332013-88332109 | - | intergenic | 5p | UGUGAAUUAGUGAGAAGACAGG | - | 75.692 | 930.292 |
| N73 | | LA | | chr14:94579976-94580053 | + | intronic (IFI27) | 3p | UUUUGUGUGUCAGGGUGCAGGU | 97.666 | 1797.692 | - |
| | | | | | | | 5p | AGGCCUCCAGACACACCGCAGC | - | 302.77 | - |
| N14 | | LA | | chr15:48873175-48873245 | - | intronic (FBN1) | 5p | UUGCUGUGAUGAUGUUCUGAGC | 97.666 | 1211.076 | 763.372 |
| N76 | | LA | | chr16:81567501-81567576 | + | intronic (CMIP) | 3p | UGAGGUGACCGCAGAUGGGA | 644.598 | 1322.184 | 359.876 |
| N17 | | | EC | chr17:504667-504745 | - | intronic (VPS53) | 3p | AGUGAUGAUGAGAGAGGCUGAAC | - | 3040.856 | 1825.452 |
| N79 | EE | LA | EC | chr19:13051290-13051374 | + | intronic (CALR) | 5p | UUGGAGGGUGUGGAAGACAUC | 1330.124 | 1131.164 | 863.192 |
| N18 | EE | | | chr19:35652419-35652495 | + | intronic (FXYD5) | 5p | UCAUGUCUGAACCAAUGAGAGC | 976.664 | 1059.692 | 654.318 |
| N80 | EE | | | chr19:52303187-52303274 | + | intronic (FPR3) | 5p | UAGCCCAUGGCUCGAUCCUCAG | 781.33 | - | - |
| N33 | EE | | | chr19:55837136-55837213 | + | intergenic | 3p | GAGAGGGGAUGUAGACGUCUAG | 1171.996 | - | - |
| N81 | EE | | | chr19:55995548-55995622 | - | intergenic | 5p | CCCCGGGGAGCCCCGCGGGG | 1315.852 | 7874.034 | 3535.15 |
| N87 | EE | | | chrX:47666470-47666558 | + | intergenic | 5p | AGGUAGGAUUAGAUCAUUAGC | 511.586 | 79.296 | 114.246 |
*EE, early EPC. LA, late EPC. EC, mature endothelial cell.
