# Supplementary material for: miRNome traits analysis on endothelial lineage cells discloses biomarker potential circulating microRNAs which affect progenitor activities
Source: BMC Genomics. 2014 Sep 18;15(1):802. doi: 10.1186/1471-2164-15-802 (PMC4176563; doi:10.1186/1471-2164-15-802)
Supplement: Supplementary file 6 — Additional file 6: Figure S1: RT-qPCR validation showing the repression of CXCR4 levels in late EPCs overexpressing with miR-221 or miR-222 (n=3). (PDF 145 KB) [file 12864_2014_6478_MOESM6_ESM.pdf]

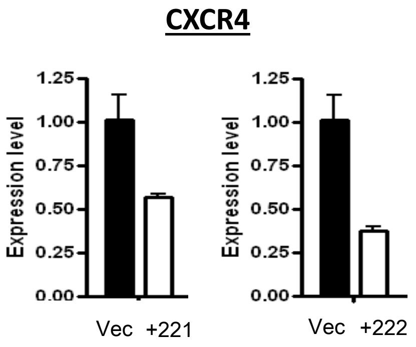

Supplementary Figure 1. RT-qPCR validation showing the repression of CXCR4 levels in late EPCs overexpressing with miR-221 or miR-222 (n=3).
